# Butyric acid fermentation from pretreated and hydrolysed wheat straw by an adapted Clostridium tyrobutyricum strain

**DOI:** 10.1111/1751-7915.12304

**Published:** 2015-07-31

**Authors:** G N Baroi, I Baumann, P Westermann, H N Gavala

**Affiliations:** 1 Department of Chemistry and Bioscience, Aalborg University (AAU)A.C. Meyers Vænge 15, DK 2450, Copenhagen, SV, Denmark; 2 Department of Chemical and Biochemical Engineering, The Technical University of DenmarkSøltøfts Plads, bldg. 229, Kgs. Lyngby, DK 2800, Denmark

## Abstract

Butyric acid is a valuable building-block for the production of chemicals and materials and nowadays it is produced exclusively from petroleum. The aim of this study was to develop a suitable and robust strain of *Clostridium tyrobutyricum* that produces butyric acid at a high yield and selectivity from lignocellulosic biomasses. Pretreated (by wet explosion) and enzymatically hydrolysed wheat straw (PHWS), rich in C6 and C5 sugars (71.6 and 55.4 g l^−1^ of glucose and xylose respectively), was used as substrate. After one year of serial selections, an adapted strain of *C. tyrobutyricum* was developed. The adapted strain was able to grow in 80% (v v^−1^) PHWS without addition of yeast extract compared with an initial tolerance to less than 10% PHWS and was able to ferment both glucose and xylose. It is noticeable that the adapted *C. tyrobutyricum* strain was characterized by a high yield and selectivity to butyric acid. Specifically, the butyric acid yield at 60–80% PHWS lie between 0.37 and 0.46 g g^−1^ of sugar, while the selectivity for butyric acid was as high as 0.9–1.0 g g^−1^ of acid. Moreover, the strain exhibited a robust response in regards to growth and product profile at pH 6 and 7.

## Introduction

Butyric acid is a four-carbon chain fatty acid with a broad range of applications in food and beverage, cosmetics and pharmaceutical industries (Zhang *et al*., 2009; Dwidar *et al*., 2012). It is currently produced from petroleum by oxidation of butyraldehyde obtained from oxosynthesis or hydroformylation of propylene (Playne, 1985) and its annual production amounts to 50 000 ton (Sauer *et al*., 2008). Development of cost-effective production of butyric acid from 2^nd^ generation biomass can reduce the use of fossil resources and, therefore, is a step towards a sustainable society. Among the key factors (e.g. feedstock availability, feedstock pretreatment, product separation and purification) in developing a cost-efficient biological production process are appropriate strain selection and development, and the use of cheap feedstocks.

*Clostridium tyrobutyricum*, a gram-positive, anaerobic, spore-forming bacterium, is a promising bacterium for biological production of butyric acid as it is characterized by higher selectivity (expressed as mass of target acid per total mass of acids produced) and higher tolerance to butyric acid compared with other bacterial species but also with the ability to catabolize both glucose and xylose, two abundant sugar monomers present in pretreated lignocellulosic biomasses (Zhang *et al*., 2009; Dwidar *et al*., 2012). The major metabolic end-products of *C. tyrobutyricum* are butyric acid, acetic acid, hydrogen and carbon dioxide. The maximum theoretical yield of butyric acid from glucose and xylose is 0.49 g g^−1^ of sugar according to the stoichiometric Eqs 1 and 2:

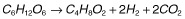
1

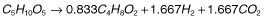
2

However, butyric acid yield in fermentations is considerably lower than the theoretical maximum as butyric acid production is accompanied by acetic acid generation. Attempts to increase the butyric to acetic acid ratio (increasing the selectivity for butyric acid) have focused on constructing mutants with inactivated *ack* and *pta* genes, which encode acetate kinase and phosphotransacetylase, respectively, in the acetate-producing pathway (Zhu *et al*., 2005; Liu and Yang, 2006; Liu *et al*., 2006a,b). Inactivation of the abovementioned genes resulted indeed in increased selectivity for butyric acid production from glucose and xylose (Zhu *et al*., 2005; Liu and Yang, 2006; Liu *et al*., 2006a,b). Also, an increased tolerance to butyric acid concentration in the fermentation broth has been reported (Zhu *et al*., 2005; Liu *et al*., 2006b). On the other hand and as anticipated, knocking out of those genes had an adverse effect on the microbial growth rate (Zhu *et al*., 2005; Liu and Yang, 2006; Liu *et al*., 2006a,b).

Fermentative butyric acid production has mainly been investigated in synthetic growth media with glucose, xylose and/or sucrose added as carbon sources (Zigova and Sturdik, 2000). During the last decade and as the concept of sustainable production of fuels and chemicals from residual feedstocks has come in the forefront, butyric acid production from 2^nd^ generation biomasses, i.e. corn fibre (Zhu *et al*., 2002; Liu *et al*., 2013), cane molasses (Jiang *et al*., 2009), Jerusalem artichoke (Huang *et al*., 2011), wheat straw, rice hull, corn stover and switch grass (Liu *et al*., 2013) has been investigated. Exploiting lignocellulosic feedstocks for the biological production of fuels and chemicals requires pretreatment and enzymatic hydrolysis to release the sugar monomers glucose and xylose from the lignocellulosic matrix. Pretreatment processes also release toxic compounds, depending on the raw material and harshness of the pretreatment, such as carboxylic acids, furan derivatives [furfural and 5-hydroxymethylfurfural (5-HMF)] and phenolic compounds, which inhibit microbial metabolism and growth (Martin and Jonsson, 2003; Alvira *et al*., 2010; Bellido *et al*., 2011). Therefore, inhibition might be one of the first obstacles to overcome when hydrolysates from 2^nd^ generation biomasses are used for biological production processes, especially when non-diluted hydrolysates with high sugar concentrations are used.

Adaptive laboratory evolution can be applied as a convenient, non-intrusive, natural tool to alleviate such inhibitory effects. The desired outcome are cultures showing improved traits, where the hydrolysate is metabolized to VFAs with increased titres and productivity by means of a stable fermentation process. At the genomic level, the aim of the adaptation process is a gain of function where genomic mutations result in new beneficial alleles that increase the cellular fitness. Adaptive laboratory evolution has been reported as a strategy for inducing microbial adaptation, metabolic regulation towards a desirable product and environmental tolerance (Portnoy *et al*., 2011; Wu *et al*., 2014). Environmental tolerance refers to adaptation to environmental factors, such as temperature, pH but also inhibitory chemical substances and takes place through gradually increased exposure of the microbial strain to a selection pressure. The advantage of adaptive selection is that natural strains are developed, which can be handled and used without the precautions necessary to handle a genetically modified microorganism. Adaptive evolution has been very effectively used for creating acid-resistant lactic acid bacteria (Wu *et al*., 2014). Also, adaptive evolution of an *Escherichia coli* strain after exposure to gradually increasing ethanol concentration for ∼ 350 generations resulted in improved ethanol tolerance (Wang *et al*., 2011). It has also been reported that *C. tyrobutyricum* could tolerate higher butyric acid concentrations after adaptation in a fibrous-bed bioreactor (Zhu and Yang, 2003).

The objective of the present study was to apply adaptive laboratory evolution in order to induce higher tolerance of a *C. tyrobutyricum* strain to wheat straw hydrolysate. Sugar uptake rates and butyric acid productivity, yield and selectivity were determined. The effect of pH on yield and selectivity of butyric acid was also assessed.

## Experimental procedures

### Microorganism, growth medium and feedstock

*Clostridium tyrobutyricum*, strain DSMZ 2637 (corresponding to ATCC 25755 and NCIB 10635) was obtained from Deutsche Sammlung von Microorganismen und Zellkulturen (DSMZ) as spores and cultivated in DSMZ medium 110. For subsequent transfers and storage, a simple growth medium was used which consist of (per litre): 0.2 g MgSO_4_ 7H_2_O; 0.0076 g MnSO_4_ H_2_O; 0.01g FeSO_4_ 7H_2_O; 4 g of casein hydrolysate; 1 mg of PABA, 2 μg of biotin, 1 mg of thiamine HCl (Obrien and Morris, 1971). Potassium phosphate buffer (which consist of 0.061 M K_2_HPO_4_ and 0.039 M KH_2_PO_4_) was added in inoculum preparations to maintain a pH value of 7 and batch fermentations were carried out in serum vials with rubber stopper sealed crimp-top vials. Resazurin of 0.5 mg l^−1^ and cysteine-HCl of 0.35 g l^−1^ were added as redox indicator and reducing agent respectively. Growth medium was kept anaerobic by nitrogen gas (purity 99.999%) flushing during preparation. Sterile D-glucose and D-xylose solutions or pretreated and enzymatically hydrolysed wheat straw (PHWS) were used as energy source depending on the experiment set-up as described in subsequent sections. Stock cultures were stored at −80°C in 10% glycerol and used as start-up inoculum for the adaptation and batch experiments.

PHWS was provided by Biogasol®, Denmark. The initial chemical composition of the wheat straw was (weight %): 33 cellulose, 26 hemicellulose, 35 lignin, 4 ash and 3 other compounds based on compositional analysis (Sluite *et al*., 2012). The wheat straw was processed by: (1) pretreatment, (2) enzymatic hydrolysis and (3) solid/liquid separation. The pretreatment process was carried out at 163°C for 15 min in a BioGasol Carbofrac™ 5D with sulphuric acid (1.4 wt.%) as catalyst. The pretreatment was used to release the hemicellulose into the liquid fraction and to keep the lignin in the solid fraction (Pedersen and Meyer, 2010). The pretreated wheat straw was subsequently cooled down and water was added to give a total solid content of 20 wt.% and pH was adjusted to pH 5.0 with sodium hydroxide. Enzymatic hydrolysis was carried out as described by Öhrman and colleagues (2013). Prior to the fermentation experiments, PHWS was passed through an 8 μm pore size filter to remove any remaining solids.

### Analytical methods

Sugars, 5-HMF and 2-furfural were quantified with high performance liquid chromatography with refractive index detector equipped with an Aminex HPX-87H column (Bio-Rad) at 60°C. A solution of 4 mM H_2_SO_4_ was used as eluent at a flow rate of 0.6 ml min^−1^. Approximately 1 ml of liquid sample was acidified with 30 μl of 2 M H_2_SO_4_ and centrifuged at 10 000 r.p.m. for 10 min. The supernatant was filtered through a 0.45 μm pore size filter. Acetic and butyric acids were quantified by gas chromatography (Perkin Elmer Claus 400) using flame ionization detector and a SUPELCO polar fused silica 0.53 ID column. The temperature of the injection port and detector was 240°C. The column temperature was initially set to 105°C for 3 min and then increased to 230°C in two steps: first to 130°C at 8°C min^−1^ and then to 230°C at 45°C min^−1^. The carrier gas was nitrogen at a flow rate of 13 ml min^−1^. Prior to analysis, samples were acidified with 17% H_3_PO_4_ to pH 2–3 and centrifuged at 10 000 r.p.m. for 10 min. The supernatant was collected and filtered through a 0.45 μm pore size filter. Phosphorus and nitrogen were measured according to standard methods (APHA, 2005). Total and volatile (TS, VS) solids were analysed according to standard methods (APHA, 2005).

### Chromosomal deoxyribonucleic acid (DNA) extraction

To verify the identity of the strains during the selection, DNA from wild-type (DSMZ 2637) and adapted cells, was isolated and analysed in the following way: Cells were grown in DSMZ medium 110. Fifteen millilitres of cell suspension was centrifuged for 1 min at 10 000 r.p.m. and the supernatant was decanted. The cell pellet was washed once in 15 ml 1xTE pH 8 and re-suspended in 100 μl TE. Fifty microlitres of 10% SDS solution was added and the suspension was whirl mixed and incubated for 30 min in an Eppendorf thermomixer set to 65°C and 300 r.p.m. The cell lysate was spun down and the supernatant was discarded. The cell pellet was then subjected to 750 W micro-wave for 3 × 1 min and dissolved in 200 μl TE by shaking for 15 min with an equal volume of chloroform–isoamyl alcohol–phenol 24:1:25. The aqueous phase was recovered by centrifugation for 5 min at 14 000 r.p.m. DNA was precipitated by adding an equal volume of 96% ethanol and centrifugation at 5°C for 30 min at 14 000 r.p.m.

The primers used were targeting 16S ribosomal DNA genes in most eubacteria (Weisburg *et al*., 1991). The forward primer sequence (fD1-B all) was: 5′ AGAGTTTGATCCTGGCTCAG. The reverse primer sequence (rP2-B) was 5′ ACGGCTACCTTGTTACGACTT. The product to be sequenced was synthesized in 2 Polymerase Chain Reactions: 1^st^ with genomic DNA from the fermenting cells as template, and a 2^nd^ using the PCR product from the 1^st^ reaction as template. The PCR thermocycler (Biometra T3000) was set to run the programme: 95°C for 2 min; (95°C for 30 s, 59°C for 30 s, 72°C for 30 s) × 30 cycles; 72°C for 5 min. The PCR product from the 2nd reaction was spin-column (Qiagen) purified. Sequencing was done by StarSEQ GmbH, Germany. Query sequence for BLAST search at National Center for Biotechnology Information entered are 300 bp–360 bp. The sequencing provided high-quality reads for queries of 360 bases (wild type) and 120 bases (adapted) respectively.

### Fermentation reactor operation

Batch experiments were performed in a 3 l Applikon® autoclavable glass reactor equipped with controllers for pH, temperature and agitation. Sterilization of medium was performed by autoclaving at 121°C for 30 min and gas sterilization was carried out using a 0.2 μm Midistar® 2000 polytetrafluoroethylene, PTFE, gas filter. Fermentations were carried out at 37°C with agitation at 150 r.p.m. and pH was maintained at 7 with sterile 4 M KOH. Prior to fermentation, the controller was calibrated for pH and temperature. To remove oxygen, sterile nitrogen gas was sparged through the reactor. Outlet gas from the reactor was passed through a condenser on top of the reactor to minimize loss of culture medium. The pressure in the reactor was assumed to be 1 atm.

### Adaptation

The first phase of the adaptation process was conducted by transferring *C. tyrobutyricum* DSMZ 2637 to gradually higher PHWS concentrations in 100 ml serum vials. Prior to fermentation, the pH of the hydrolysate was adjusted to 7 by using 5 M NaOH. Subsequently the hydrolysate was centrifuged at 10 000 g and the supernatant was filtered using a sterile blue cap bottle-top filter (Nalgene, pore size 0.45 μM) and subsequently flushed with sterile N_2_ gas (99.999% purity). Batch fermentation was conducted in 24 ml hydrolysate/growth medium mixture in a 100 ml rubber stopper sealed crimp-top vial with a 4% (v v^−1^) inoculation.

The hydrolysate concentration in which the cells were able to grow was defined prior to the adaptation series. Vials containing 2%, 5%, 10%, 25%, 50% and 100% (v v^−1^) PHWS hydrolysate were inoculated with a grown culture of *C. tyrobutyricum* and growth was monitored by measuring gas pressure in the head space. Growth was observed in the vial with 10% PHWS, and subsequently, vials containing 12.5%, 15%, 17.5%, 20% and 22.5% PHWS were inoculated with a grown culture of *C. tyrobutyricum*. Finally, the 10% PHWS was chosen as a starting PHWS concentration for the subsequent adaptation steps, as hesitant growth was observed in the vials containing 12.5% PHWS after a long lag phase.

From a pre-culture in growth phase, which was prepared in hydrolysate/medium mixture, 1 ml (4% v v^−1^) inoculum was added to the fermentation broth and incubated at 37°C. The vials were stirred manually daily. To ensure a transfer of growing cells, the outgrown culture at a specific PHWS concentration was left for approximately 10–14 days to promote adaptation and then re-inoculated at the same hydrolysate concentration. When cell growth had resumed, an inoculum was transferred to vials containing substrate with a higher hydrolysate concentration. After a lag phase of 2–4 days, growth became clearly visible in the dark brown hydrolysate and gasses were released from the vials. The following days, cultures were checked for further gas production and as it ceased they were left untouched for approximately one week stationary phase. For every fermentative round, the hydrolysate concentration was increased in linear increments normally of around 2.5 g l^−1^ in order to reduce lag phase and promote growth in the freshly inoculated fermentation vials.

Keeping a stable pH in 100 ml vials by using buffer was impossible when the PHWS concentration increased above 40%. The second phase of the adaptation was, therefore, carried out in the 3 l bioreactor with a working volume of 1000 ml and with a 10% (v v^−1^) inoculum. The composition of the growth medium was simplified by omitting cysteine-HCl and resazurin. Successive batch fermentations were carried out with increasing concentrations of PHWS from 40% to 80% (v v^−1^). Once approximately half of the sugars were consumed, cells were transferred to a fresh batch of substrate with increased concentration of PHWS. The adapted strains were stored at −80°C in 10% glycerol after each batch cycle.

### Batch fermentations

Three sets of batch fermentations were performed with the adapted *C. tyrobutyricum* strain: (i) fermentation with continuous flushing of the headspace of the fermentor with nitrogen, (ii) fermentation with pulse feeding and (iii) fermentation at different pH values. The experiments were performed in the 3-L Applikon® reactor with a working volume of 1 l at 37°C, pH 7, agitation speed set at 150 r.p.m. and a 10% inoculum. The 1^st^ experiment was performed with 70% (v v^−1^) PHWS and nitrogen gas was supplied continuously in the gas phase at a rate of 2 ml s^−1^. The second experiment was started with 5% (v v^−1^) PHWS and after almost all glucose and xylose had been consumed, subsequent feedings of 50 ml 100% PHWS took place. The third experimental series was carried out with *C. tyrobutyricum* growing in 5% PHWS and at three different pH values (5, 6 and 7).

### Stoichiometric calculations

Stoichiometric calculations were based on product yields, and calculation of the glucose and xylose electron equivalents were partitioned between energy production (catabolism of glucose and xylose to various products) and biomass synthesis (Rittmann and McCarty, 2001). Assuming glucose and xylose as the sole electron donors in our experiments, and calculating the fraction of electron equivalents found in each of the products, the theoretical energy reaction was constructed. The organic half reactions used for the substrates (glucose and xylose) and products (hydrogen, butyric and acetic acids) are as following (Eqs 3–7):

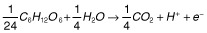
3

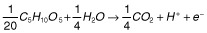
4

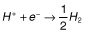
5

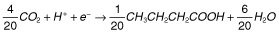
6

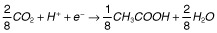
7

The fraction of the electron donors’ electron equivalents used for energy production (f_e_) was calculated from the difference between the product yields predicted by the theoretical energy reaction and the actual measured yields as reported by Antonopoulou and colleagues (2011). The fraction of the electron donors’ electron equivalents used for cell synthesis (f_s_) was then calculated using Eq. 8 (Rittmann and McCarty, 2001):

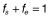
8

## Results and discussion

The composition of the PHWS used for the adaptation and fermentation experiments in the present study is shown in Table 1. PHWS was rich in glucose and xylose (127 g l^−1^ in total) and it also contained small amounts of arabinose and cellulose. Furfural and 5-HMF generated from the pretreatment were also present, while acetic acid concentration was considerable, at 7 g l^−1^.

**Table 1:** Composition of PHWS

Name of compounds	Amount
Glucose (g l^−1^)	72 ± 0.2
Xylose (g l^−1^)	55 ± 0.2
Arabinose (g l^−1^)	4.2 ± 0.0
Cellobiose (g l^−1^)	2.3 ± 0.5
Acetate (g l^−1^)	7.1 ± 0.0
5-HMF (g l^−1^)	0.02 ± 0.03
2-Furfural (g l^−1^)	0.18 ± 0.04
Total inorganic phosphorous (mg l^−1^)	200 ± 4
Total inorganic nitrogen (g l^−1^)	0.11 ± 0.00
TS %(g g^−1^)	12 ± 0.0
VS % (g g^−1^)	6.4 ± 0.1
pH	5.0

### Adaptation – 1^st^ phase

As wild-type *C. tyrobutyricum* cells did not grow in 100% PHWS, a laboratory adaptation was applied as a strategy to increase tolerance to PHWS. The level of initial tolerance was established by growing cells at different concentrations of PHWS. The cultures were monitored for growth and cells growing at the highest concentration of PHWS were transferred to vials with fresh medium. The initial tolerance was found to be at 10% (v v^−1^) PHWS. Then a succession of serial cell transfers was applied to vials where the PHWS concentration was gradually increased from initial 10% (v v^−1^) PHWS containing 13 g l^−1^ of sugar and 27 mg l^−1^ of HMF and furfural to 60% containing 76 g l^−1^ of total sugar (glucose and xylose) and 163 mg l^−1^ of HMF and furfural (Table 2). In the fifth and sixth experiments, the adapted *C. tyrobutyricum* consumed 28% and 20% of the glucose content; however, xylose remained unmetabolized. At PHWS concentrations above 40% (fifth and sixth experiment) the pH eventually dropped below 4.5 in the serum vials. Therefore, adaptation at higher concentrations of PHWS was carried out in the 3 l fermentor where the pH could be controlled.

**Table 2:** Initial total sugar (glucose and xylose) and HMF and furfural concentrations in the six fermentation rounds with gradually increased amount of PHWS during adaptation phase I

Fermentation round	Total sugars (g l^−1^)	HMF and furfural (mg l^−1^)
1	13	20
2	15	23
3	29	46
4	43	68
5	61	96
6	76	120

### Adaptation – 2^nd^ phase

Batch fermentations in the 3 l fermentor started with 40% (v v^−1^) PHWS and were continued up to 80%. Due to the risks of contamination in the complex fermentor experiments with mono-cultures, 16S ribosomal ribonucleic acid, rRNA, genes amplified by PCR from both wild-type and adapted strains were sequenced and aligned to the genome database at GenBank. In all cases, the PCR products matched 100% to the corresponding 16S ribosomal RNA gene of *C. tyrobutyricum*.

**Figure 1:**
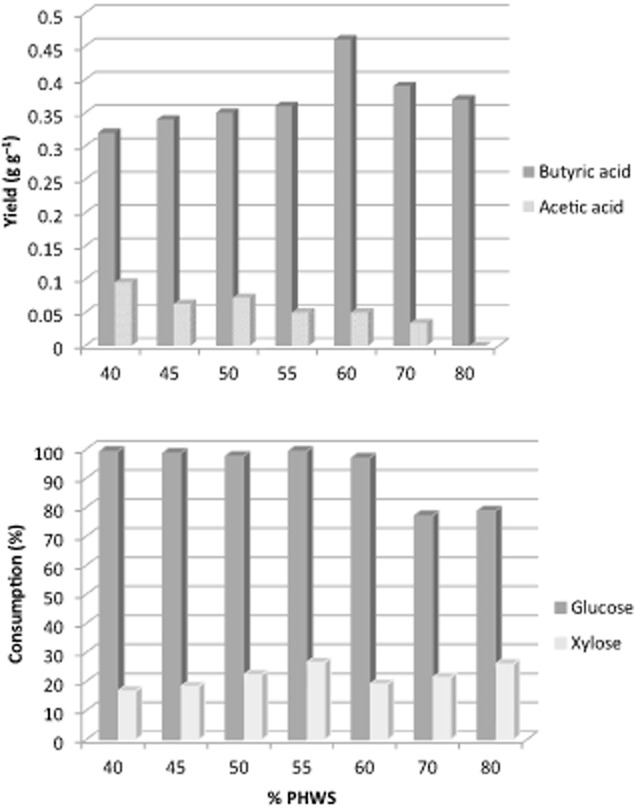
Butyric and acetic acid yields and sugar consumption obtained with increasing concentrations of PHWS during the 2^nd^ adaptation phase of *C. tyrobutyricum*.

Butyric and acetic acids were the major products observed during the adaptation and the yields as well as sugar consumption are shown in Fig. 1. Glucose was the major sugar consumed while only minor amounts of xylose and arabinose were metabolized. The apparent yield of butyric acid was in the range of 0.32–0.46 g g^−1^ of sugar with the highest yield obtained at 60% PHWS fermentation. The butyric acid yield at 60–80% PHWS fermented was comparable and even higher than the yields achieved with mutants with inactivated *ack* and *pta* genes, 0.42 (Liu *et al*., 2006b) and 0.38 g g^−1^ of glucose (Zhu *et al*., 2005; Liu and Yang, 2006) respectively. Acetic acid apparent yield was decreasing with increasing PHWS concentration leading to increasing selectivity towards butyric acid, which lied between 0.78 and 1 g g^−1^ of acid. The very low and especially the ‘zero’ net yield of acetic acid during the fermentation with 80% PHWS could be an indication of acetic acid uptake to form butyric acid. Such an activity has been reviewed by Zhang and colleagues (2009) and reported for *C. tyrobutyricum* in the study of Michel-Savin and colleagues (1990). Also, *Clostridium thermobutyricum* has shown such potential (Canganella *et al*., 2002).

**Figure 2:**
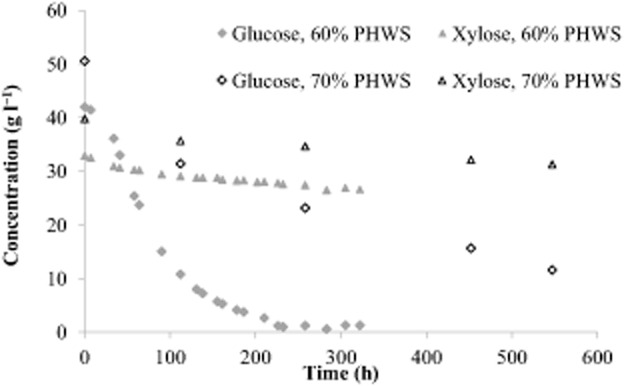
Glucose and xylose utilization during batch fermentations of 60% and 70% PHWS by adapted *C. tyrobutyricum*.

Despite that butyric acid yield and selectivity were high, the sugar consumption rates and consequently the butyric acid productivity were low, especially at elevated concentrations of PHWS. In Fig. 2 glucose and xylose consumption during batch fermentations of 60% and 70% PHWS are shown. The average glucose consumption rates were 210 and 71 mg l^−1^ h^−1^ at 60% and 70% PHWS, respectively, while xylose consumption rates were even lower, 19 and 15 mg l^−1^ h^−1^ respectively. The fermentation of 70% PHWS was repeated applying continuous flushing with nitrogen gas in order to remove hydrogen and thereby increase the consumption rates of the sugars. Average sugar consumption rates, butyric and acetic acid productivities and yields are compared in Table 3.

**Table 3:** Characteristics of the batch fermentations with and without flushing with nitrogen gas

	70% PHWS No flushing with N_2_^a^	70% PHWS Flushing with N_2_^b^
Average glucose consumption rate, g l^−1^ h^−1^	0.07	0.51
Average xylose consumption rate, g l^−1^ h^−1^	0.02	0.07
Average acetic acid production rate,^c^ g l^−1^ h^−1^	0.003	0.05
Acetic acid yield,^c^ g g^−1^ sugars	0.03	0.08
Acetic acid concentration,^d^ g l^−1^	7.2	10.4
Average butyric acid production rate, g l^−1^ h^−1^	0.03	0.21
Butyric acid yield, g g^−1^ sugars	0.40	0.33
Butyric acid concentration, g l^−1^	19	20
Butyric acid selectivity, g g^−1^ acids	0.92	0.80

a Calculations done at the time when glucose was consumed (at 232 h).

b Calculations done at the time when glucose was consumed (at 100 h).

c Yield and production rate of acetic acid was calculated based on the amount metabolized by the strain.

d Total acetic acid (from PHWS and biologically produced).

Flushing with nitrogen gas affected the sugar consumption rates positively as glucose and xylose consumption rates increased seven- and fivefold compared with the fermentation without flushing. Similarly, butyric acid production rate increased while the selectivity for butyric acid decreased from 0.92 to 0.80. This is consistent with the observations in the study of Canganella and colleagues (2002) where removal of hydrogen from the headspace of glucose fermentation by *C. thermobutyricum* resulted in decreased yield of butyric acid while acetic acid yield was positively affected. Mizuno and colleagues (2000) have also reported that the distribution of fermentation products depends on the hydrogen partial pressure in the headspace of the reactor. Decreased ratios of butyric to acetic acid due to the removal of hydrogen gas were also reported by van Andel and colleagues (1985) during continuous fermentation of glucose by *Clostridium butyricum*. The increased rates of glucose and xylose consumption could be attributed to the increased electron channelling through the Nicotinamide adenine dinucleotide, NADH–ferredoxin oxidoreductase system at low hydrogen partial pressures. This also influenced the yields of metabolic products: lower hydrogen partial pressure favoured ATP production through the acetic acid pathway where 4 moles H_2_ are produced per mole acetic acid (compared with 2 moles H_2_ per mole butyric acid) and hence acetic acid yield increased when flushing was applied. In other words, low hydrogen partial pressures favour balancing of electron equivalents in the cell through H_2_ generation.

**Figure 3:**
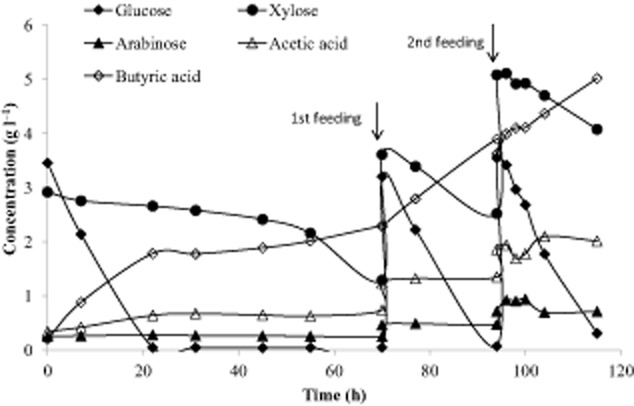
Sugar consumption and acid production profiles during batch fermentation with two consecutive pulse feedings of PHWS.

As shown in Fig. 2 and Table 3, glucose was consumed at a much higher rate than xylose during fermentation of 60% and 70% PHWS. However, Zhu and colleagues (2002) have reported simultaneous consumption of glucose and xylose at comparable rates by *C. tyrobutyricum* from corn fibre hydrolysate during fed-batch fermentations. In that study, the initial concentration of glucose and xylose at each cycle was 15 g l^−1^, which is much lower than the sugar concentrations found at 60% PHWS (43 and 33 g l^−1^ of glucose and xylose respectively). The concentration difference could play a significant role, especially if a mechanism for autoregulatory limitation of sugar consumption is active in *C. tyrobutyricum* (Bruckner and Titgemeyer, 2002). A fed-batch experiment with PHWS and at an initial sugar concentration corresponding to 5% PHWS was then performed. Sugar and acid concentration profiles are shown in Fig. 3.

During the first 70 h, the strain exhibited a diauxic growth pattern, consuming first glucose and subsequently xylose. During this period, xylose consumption did occur at some extent, although at a very low rate. The average rate of xylose consumption during the first 45 h was 0.011 g l^−1^ h^−1^, which is comparable with the consumption rates observed for xylose in 60% and 70% PHWS fermentations. Glucose consumption occurred at an average rate of 0.16 g l^−1^ h^−1^. After xylose consumption was established, the first feeding with 5% PHWS was performed after which glucose and xylose were consumed in parallel at the subsequent feedings. Glucose and xylose average consumption rates during the two feedings were 0.14 and 0.05 g l^−1^ h^−1^ respectively. In most *Clostridia*, the glucose uptake is constitutive while xylose transport and metabolism is inducible, which is often reflected in the substrate preference where the substrates for which the cellular mechanisms are already in place are consumed first (Mitchell *et al*., 1995). The glucose repression observed during the diauxic growth observed at the beginning of this experiment was, however, only initial. As soon as xylose metabolism was induced, both mechanisms ran in parallel. In a study of mixed glucose and xylose fermentation in *C. acetobutylicum*, Ounine and colleagues (1985) saw the same metabolic pattern and concluded that the synthesis of the xylose transport system was repressed by the initial presence of glucose in the medium. After the xylose transport system had been induced, the lower uptake rate compared with glucose was due to a lower affinity for xylose than for glucose. We anticipate that a similar mechanism is responsible for the observed metabolic pattern in Fig. 3.

### Effect of pH on yield and selectivity

**Figure 4:**
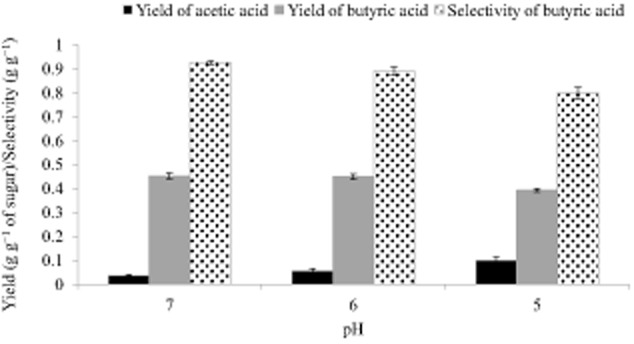
Yields of butyric and acetic acids and selectivity of butyric acid under different pH values.

Figure 4 shows that there was not any significant difference (*p* = 0.797, student’s T-test, tails-2, type-2 ) in butyric acid yields at pH 6 and 7, which were 0.45 ± 0.01 and 0.45 ± 0.01 g g^−1^ respectively. Butyric acid selectivity was high, 92.5 ± 1% and 88.9 ± 1.8% at pH 7 and pH 6 respectively. On the other hand, the yield of butyric acid at pH 5 was 0.39 ± 0.01 g g^−1^ which was significantly lower (*p* value less than 0.002) compared with the yields obtained at pH 6 and pH 7. As anticipated, the selectivity at pH 5 was also lower (79.8 ± 2.4%) in comparison with those obtained at pH 6 and pH 7. This could be attributed to lower phosphotransacetylase (PTA) activity, observed during the growth of *C. tyrobutyricum* on a synthetic, xylose-based growth medium, which results in elevated acetic acid yields (Zhu and Yang, 2004).

Although other studies have reported pH values of 5.8 (Michel-Savin *et al*., 1990) and 6.3 (Jo *et al*., 2008) as optimal for butyric acid yield and selectivity by *C. tyrobutyricum*, our adapted *C. tyrobutyricum* strain on PHWS exhibited similar butyric acid yield at both pH values of 6 and 7.

Stoichiometric equations representing the energy reactions for the experiments at different pH values are shown in Table 4. The electron fraction f_e_ is higher at pH values of 6 and 7, while a lower f_e_ value characterized the experiment at pH 5. At a first glance this seems controversial to what was anticipated as maintenance requirements of the microbial cells are higher at pH 5 than at pH 6 and 7 and therefore, a higher value of f_e_ would be expected. However, the cell compensated for the increased energy requirements by shifting the energy reaction to the more energetically beneficial component, acetic acid as it is reflected by the increased stoichiometric coefficient. Apparently, shifting the catabolic reactions to more energetically favourable metabolites was a preferable strategy for the cells as they could still maintain a high yield of microbial biomass under non-favourable growth conditions.

**Table 4:** Stoichiometric coefficients for the overall reaction applied for *C. tyrobutyricum* growth on 5% PHWS at different values of pH

pH	Reactants				Products						Electron fractions	
	C_6_H_12_O_6_	C_5_H_10_O_5_	HCO_3_^−^	NH_4_^+^	C_5_H_7_O_2_N	H_2_	CH_3_CH_2_CH_2_COOH	CH_3_COOH	H_2_O	CO_2_	f_e_	f_s_
7	1	0.90	0.29	0.29	0.29	1.11	1.61	0.19	2.24	2.46	0.86	0.14
6	1	0.94	0.27	0.27	0.27	1.08	1.64	0.30	2.19	2.45	0.87	0.13
5	1	0.86	0.39	0.39	0.39	0.89	1.38	0.51	2.47	2.21	0.81	0.19

## Conclusions

The purpose of this work was to develop a *C. tyrobutyricum* strain capable to grow in concentrated wet exploded and enzymatically hydrolyzed wheat straw, PHWS. The developed strain was able to grow in 80% (v v^−1^) PHWS compared with an initial tolerance to less than 10% PHWS. It is noticeable that the adapted *C. tyrobutyricum* strain was characterized by a high yield and selectivity to butyric acid. Specifically, the butyric acid yield at 60–80% PHWS ranged between 0.37 and 0.46 g g^−1^ of sugar, while the selectivity for butyric acid was as high as 0.9–1.0 g g^−1^ of acid. Moreover, the strain was capable of simultaneous utilization of both glucose and xylose and it exhibited a robust response in regards to growth and products profile at pH 6 and 7. The developed strain showed potential for industrial exploitation; however, the drawback was the low consumption rates of sugars. Therefore, a suitable process configuration allowing for higher sugar consumption rate and butyric acid productivity should be developed and applied.

## Conflict of interest

None declared.
